# The Association of Prepregnancy Body Mass Index with Pregnancy Outcomes in Chinese Women

**DOI:** 10.1155/2022/8946971

**Published:** 2022-03-26

**Authors:** Jing Zhang, Wensheng An, Li Lin

**Affiliations:** Division of Gynecology and Obstetrics, Peking University International Hospital, Beijing, China

## Abstract

Our study was to evaluate the association between prepregnancy body mass index (BMI) and pregnancy outcomes. A total of 1546 women who attended prenatal care clinics and delivered at the Peking University International Hospital, Beijing, China, from October 2018 to April 2020 was included. This research explored gestational, perinatal, and postpartum outcomes, including gestational diabetes, anemia, preeclampsia, preterm premature rupture of membranes (PPROM), and postpartum hemorrhage. Participants were divided into underweight (BMI < 18.5 kg/m^2^), normal weight (18.5 kg/m^2^ ≤ BMI ≤ 23.9 kg/m^2^), overweight (24 kg/m^2^ ≤ BMI ≤ 27.9 kg/m^2^), and obese (BMI ≥ 28 kg/m^2^) groups. Logistic regression analysis was used to analyze the association between prepregnancy BMI and pregnancy outcomes, and odds ratio (OR) with 95% confidence interval (95% CI) was calculated. After adjusting potential confounders, the risk of PPROM was higher in the underweight group than the normal weight group (OR = 1.864, 95% CI: 1.269-2.737, *P* < 0.01). Prepregnancy obesity was associated with higher odds of gestational diabetes (OR = 2.649, 95% CI: 1.701-4.126, *P* < 0.001) and preeclampsia (OR = 3.654, 95% CI: 1.420-9.404, *P* < 0.01) than the normal weight group, whereas it correlated with the lower risk of anemia (OR = 0.300, 95% CI: 0.128-0.704, *P* < 0.01). Our findings may provide evidence for the importance of keeping normal weight for Chinese women when preparing for pregnancy.

## 1. Introduction

Body mass index (BMI), categorized into underweight, normal weight, overweight, and obesity, is a prominent indicator to measure several health conditions [1–3]. For women at reproductive age, preconceptional body weight influences gestational, perinatal, and postpartum outcomes and even the child's health [4–6]. When conceiving with an abnormal BMI, women are prone to have adverse pregnancy outcomes, like abnormal fetal growth, enhancing the risk of macrosomia or small for gestational age (SGA) births, which poses long-term implications for child health [7, 8].

The substantial proportion of overweight and obese individuals worldwide has led to a vast research endeavor. Globally, approximately 1.9 billion adults were overweight, and 609 million were obese in 2015 [9]. Excessive weight, once a health problem in developed countries, is now affecting several developing countries. In China, the adult overweight rate had raised from 27.8% in 2010 to 33.5% in 2016 and the obese rate raised from 5.4% in 2010 to 7.0% in 2016 [10]. The dramatic growth of the overweight and obese population in low-income and middle-income countries (LMICs) is accompanied with increasing overweight and obese women at reproductive age [11]. Prepregnancy overweight and obesity are linked with ovulatory dysfunction, which may lead to longer conception time and infertility [12]. The incidence of gestational diabetes is also strongly associated with prepregnancy body weight [13]. Moreover, excessive preconceptional weight precedes gestational obesity, increasing the risk of preterm premature rupture of membranes (PPROM), cesarean delivery, postpartum hemorrhage, and preeclampsia [14–16].

On the other hand, there were approximately 462 million underweight adults worldwide in 2014, according to the World Health Organization [17]. In developed countries, the prevalence of underweight is considerably lower than overweight and obesity and is showing a decreasing trend. In the United States, the prevalence of underweight was 3.6% in 2015, which decreased 8% as compared to the prevalence in 2011 [18]. However, underweight and malnutrition, particularly maternal and child malnutrition, remain prevalent in LMICs [19]. In China, the prevalence of underweight women at reproductive age in rural regions was 7.8% in 2016 [20]. Though less prevalent than overweight and obesity, underweight is commonly associated with malnutrition, which restricts fetal growth and contributes to 12% of neonatal death worldwide [19, 21]. Prepregnancy underweight also increases the risk of several adverse maternal and child health conditions, such as SGA births, anemia, and preterm birth [18, 22], as the result of malnutrition.

The association between preconceptional weight, particularly excessive weight, and pregnancy outcomes has been established in high-income countries. Nevertheless, limited studies have explored such relationship among the Chinese population. Genetic, environmental, and dietary differences in China may impact the generalizability of findings from previous studies to the Chinese population. Therefore, we conducted this research in an attempt to examine the relationship between prepregnancy BMI and pregnancy outcomes among Chinese women at reproductive age.

## 2. Materials and Methods

### 2.1. Study Population and Data Source

In this cohort study, data of 1546 women who attended prenatal care clinics and delivered at the Peking University International Hospital, Beijing, China, from October 2018 to April 2020 was included. This research was approved by the Ethnics Review Board of Peking University International Hospital (2021-024BMR). The body weight and height before pregnancy were self-reported by mothers at the first prenatal visit (at 6-8 weeks of gestation) and collected by physicians. Height was measured to the nearest 0.1 cm by a stadiometer, and weight was measured to the nearest 0.1 kg by an electronic scale. Body mass index (BMI) was calculated using the maternal self-reported prepregnancy weight and height (kg/m^2^). According to the Guidelines for Prevention and Control of Overweight and Obesity in Chinese Adults, developed by the Department of Disease Control Ministry of Health in China, the normal BMI range for the Chinese population was 18.5-23.9 kg/m^2^ [23]. Underweight, overweight, and obese were defined as BMI < 18.5 kg/m^2^, BMI between 24 and 27.9 kg/m^2^, and BMI ≥ 28 kg/m^2^, respectively. Since the study participants of this research were all Chinese, we categorized the BMI groups based on the Chinese standard.

### 2.2. Outcomes

We examined common gestational, perinatal, and postpartum outcomes in this research, including gestational diabetes, anemia, preeclampsia, PPROM, and postpartum hemorrhage, and explored the effect of prepregnancy BMI on these outcomes. Other outcomes, such as cesarean delivery, pregnancy-induced hypertension, placenta previa, and placental abruption, were not considered due to the missing information or extremely small sample size.

(i) Gestational outcomes and diagnostic criteria
  1. Gestational diabetes was diagnosed when (i) fasting blood glucose level ≥ 5.1 mmol/L; (ii) 75 g Oral Glucose Tolerance Test (OGTT) plasma glucose level ≥ 11 mmol/L after 1 hour or ≥8.5 mmol/L after 2 hours; (iii) the presence of other hyperglycemia symptoms
  2. Gestational anemia was determined when the hemoglobin concentrations < 110 g/L
  3. Preeclampsia was defined when the blood pressure exceeded 140/90 mmHg and accompanied with one or more of the following symptoms: (i) protein/creatinine ratio > 0.3; (ii) urine dipstick reading > (+); (iii) impaired coronary, pulmonary, renal, or cerebral functions in the absence of proteinuria
(ii) Perinatal outcomes
  1. PPROM was determined as spontaneous rupture of membrane at less than 37 weeks of gestations and at least 1 hour before the onset of contraction
(iii) Postpartum outcome
  1. Postpartum hemorrhage was defined as blood loss of ≥500 mL within 2 h after delivery

### 2.3. Potential Covariates

Multiple gestations were associated with an increased risk of pregnancy outcomes, such as gestational hypertension, gestational diabetes, and preterm delivery [24]. Additionally, the conception method, spontaneous pregnancy or using assisted reproductive technology (ART), had been shown to impact the risk of pregnancy outcomes [25]. Therefore, the number of pregnancies and conception method were adjusted in this research. Moreover, we included gestational diabetes as a covariate due to the fact that gestational diabetes possesses an additive impact on the risk of adverse pregnancy or delivery outcomes [26]. Also, other confounders (including PPROM, anemia, and preeclampsia) were, respectively, adjusted to eliminate the potential bias when addressing the pregnancy outcomes [27–29].

### 2.4. Statistical Analysis

Prior to analysis, variable distribution was tested for normality by the Shapiro normality test. Continuous variables were presented in mean standard deviation, while categorical variables were displayed in cases and proportions. Abnormally distributed continuous variables were described in medians and interquartile range and compared with the Mann–Whitney *U* test. Categorical variables were compared using Fisher's exact test and Pearson's chi-square. The association between prepregnancy BMI and pregnancy outcomes was analyzed using logistic regression analysis, which was also implemented to obtain the odds ratio (OR) and 95% confidence interval (95% CI). The result was considered significant when the *P* value was less than 0.05. All analyses were conducted by SAS 9.4 (SAS Institute, Inc. Cary, NC, USA).

## 3. Results

### 3.1. Study Population

Of the included 1546 women, 91.91% (*n* = 1421) had a history of pregnancy, 8.46% (*n* = 130) underwent ART, and 2.73% (*n* = 42) were multiple pregnancies (Table 1). Normal weight women constituted the greatest proportion (67.86%) of the study participants, followed by overweight (15.39%), underweight (9.96%), and obese (6.79%) women. There was a significant difference in age and conception method (both *P* < 0.001) among the underweight group, normal group, overweight group, and obese group. The significant difference was not found in history of pregnancy and number of pregnancies among the four groups, with *P* value of 0.240 and 0.291, respectively. One or more adverse gestational, perinatal, and postpartum outcomes occurred in 82.08% (*n* = 1269) of the study population, which included 23.16% (*n* = 358) gestational diabetes, 20.89% (*n* = 323) PPROM, 16.62% (*n* = 257) anemia, 7.89% (*n* = 122) postpartum hemorrhage, and 2.20% (*n* = 34) preeclampsia.

### 3.2. Main Outcome

In the logistic regression models, the normal weight group was defined as the reference group. Comparing to the reference group, the unadjusted logistic regression model revealed a significantly higher odds of PPROM in the underweight group (OR = 1.757, 95% CI: 1.210-2.549, *P* < 0.01). The obese group showed a significantly higher odds of gestational diabetes (OR = 2.838, 95% CI: 1.881-4.282, *P* < 0.001) and preeclampsia (OR = 4.472, 95% CI: 1.908-10.483, *P* < 0.01) than the normal weight group. In contrast, the odds ratio of anemia was statistically lower than that of the reference group (OR = 0.347, 95% CI: 0.159-0.760, *P* < 0.01). No statistical difference was found between overweight and adverse pregnancy outcomes. The results are summarized in Table 2.

After adjusting for age and conception method (Table 3), the similar results were found. The PPROM incidence remained higher in the underweight group than the normal weight group (OR = 1.944, 95% CI: 1.329-2.845, *P* < 0.01). The pregnancy outcomes, including gestational diabetes, PPROM, anemia, postpartum hemorrhage, and preeclampsia, were not statistically different between the overweight group and reference group. The gestational diabetes (OR = 2.758, 95% CI: 1.782-4.269, *P* < 0.001) and preeclampsia (OR = 3.512, 95% CI: 1.408-8.762, *P* < 0.01) incidence was significantly higher, while the occurrence of anemia (OR = 0.345, 95% CI: 0.156-0.760, *P* < 0.01) was significantly lower in the obese group as compared to the reference group.

Considering that multiple birth has been demonstrated as an important risk factor for developing gestational diabetes in some studies [7, 30], we further included the number of pregnancies as a covariate. In Table 4, after adjusting for age, conception method, and number of pregnancies, we found similar results. The OR for PPROM was 1.902 (95% CI: 1.296-2.790), with *P* < 0.01, in the underweight group. In the obese group, the significant difference was found in gestational diabetes, anemia, and preeclampsia, with OR of 2.726 (95% CI: 1.757-4.231, *P* < 0.001), 0.299 (95% CI: 0.128-0.698, *P* < 0.01), and 3.766 (95% CI: 1.504-9.427, *P* < 0.01), respectively. The overweight group showed no significance in the pregnancy outcomes.

Given that gestational diabetes poses an additive impact on the risk of adverse pregnancy or delivery outcomes, we included gestational diabetes as a covariate [31]. We also, respectively, adjusted the other confounders (including PPROM, anemia, and preeclampsia) to eliminate the potential bias when addressing the pregnancy outcomes [27–29]. The results were consistent with the previous analysis. In Table 5, after adjusting age, conception method, the number of pregnancies, anemia, and preeclampsia, the obese group showed a higher risk of gestational diabetes, with OR of 2.649 (95% CI: 1.701-4.126, *P* < 0.001). Adjusting for age, conception method, the number of pregnancies, gestational diabetes, anemia, and preeclampsia, the OR for PPROM was 1.864 (95% CI: 1.269-2.737, *P* < 0.01), indicating that the risk of PPROM was significantly higher in the underweight group. The risk of anemia was lower (OR = 0.300, 95% CI: 0.128-0.704) after adjusting for age, conception method, the number of pregnancies, gestational diabetes, and preeclampsia, and the risk of preeclampsia was higher (OR = 3.654, 95% CI: 1.420-9.404) after adjusting for age, conception method, the number of pregnancies, gestational diabetes, and anemia in the obese group (both *P* < 0.01).

## 4. Discussion

The results of this research indicated a significant association between prepregnancy BMI and pregnancy outcomes. After adjusting the confounders, prepregnancy BMI < 18.5 kg/m^2^ is linked with 1.864 folds of the PPROM risk compared with normal prepregnancy BMI. The risk of gestational diabetes is 2.649 times higher in participants who were obese than participants who were at normal weight before pregnancy. Moreover, preeclampsia is 3.654 times more prevalent among participants who were obese before pregnancy comparing to the normal weight participants. Interestingly, pregestational BMI ≥ 28 kg/m^2^ exerts a protective effect on anemia, with the risk of anemia decreasing 70% comparing with the normal weight group. One possible explanation is that overconsumption of food, such as red meat, [32] increases the overall nutrient intake among obese participants, which decreases the risk of iron deficiency-related anemia.

A previous cross-sectional study on Chinese women specifically investigated the relationship between BMI and anemia [33]. The findings were similar to our results, which indicated a significantly lower risk of anemia in overweight and obese women than the normal weight women. Furthermore, the anemia study also collected dietary consumption data of the study participants. The dietary analysis revealed a higher iron and vitamin C intake of the overweight and obese participants than the underweight participants, implying that higher nutrient consumption among Chinese obese and overweight women may be the cause of the discrepancy. Our research detected a similar decreased risk in the obese group but not the overweight group, possibly due to the smaller sample size and lower proportion of overweight participants in our study (*n* = 238, 15.39%) than those of the anemia study (*n* = 424, 27.6%).

A prospective cohort U.S. study examined 4500 women who attended clinics and delivered at the University of Mississippi Medical Center with some pregnancy outcomes analogous to our study, including preeclampsia and gestational diabetes [34]. The incidence of preeclampsia increased significantly in women with BMI ≥ 30 kg/m^2^, while the prevalence was not significantly different between the underweight and overweight groups. For gestational diabetes, significant increase in incidence was observed in women with BMI ≥ 25 kg/m^2^, with the overweight group indicating approximately 2-fold increase in the gestational diabetes odds and the obese group showing about 3-fold increase. Based on the severity of obesity, the cohort study further categorized the obese group using BMI ranges 30-34.9 kg/m^2^, 35-39.9 kg/m^2^, 40-44.9 kg/m^2^, and ≥45 kg/m^2^. The prevalence of preeclampsia raised up to 4.75 times at BMI ≥ 45 kg/m^2^ comparing to BMI < 30 kg/m^2^, and the odds ratio of gestational diabetes elevated to 5.98 times at BMI 40-44.9 kg/m^2^ as compared to BMI < 25 kg/m^2^.

Most of the findings of the cohort study are allied with our results, showing that prepregnancy BMI is positively associated with adverse pregnancy outcomes, including gestational diabetes and preeclampsia. The main discrepancy between our study and the cohort study is the relationship between the overweight group and the pregnancy outcomes. Our study did not detect any increase in preeclampsia and gestational diabetes incidence of the overweight group. Difference in the target population, the US population vs. Chinese population, may result in discrepancies. The disparity may also be attributed to the smaller proportion of the overweight population, which is the shortcoming of our research. In China, the adult underweight rate was 7.8%, overweight rate was 33.5%, and obese rate was 7.0% in 2016 [10, 20]. In the current research, the underweight rate (10.0%) and obese rate (6.8%) are similar to the epidemiology study. However, the overweight (15.4%) rate is 18.1% lower than the estimates, decreasing the representativeness of the overweight sample in our study. Therefore, findings of the underweight and obese women in this study may be generalized to the Chinese female population at reproductive age, whereas results of the overweight group require cautious interpretation.

This study discovers evidences to ascertain the need of maintaining a healthy weight and increasing the overall nutrient intake for Chinese women when preparing for pregnancy. It also should be acknowledged that there are some limitations in this study. First, this is a retrospective study and the prepregnancy weight is self-reported, which may appear recall error, causing an underestimation or overestimation of prepregnancy weight. Second, the study participants are from a tertiary center located in Beijing, a city rich in medical resource, which may cause selection bias. Third, besides the adverse outcomes investigated in this research, abnormal prepregnancy BMI has been linked with several other pregnancy-related variates, such as early pregnancy loss, stillbirth, abnormal birth weight, thromboembolism, induced labor, cesarean section, premature birth, and postpartum depression [11, 13, 15, 35–41]. Since limited information of the study sample and limited overweight sample were collected in this study, we investigated the variates that were available for analysis in this research. Future study may target other pregnancy-related variates and include representative sample overweight population.

## 5. Conclusions

In conclusion, underweight increased the risk of PPROM, and obesity increased the risk of gestational diabetes and preeclampsia while it decreased the risk of anemia. The findings of this study may provide evidence of the importance of maintaining a normal body weight and keeping a reasonable diet and balanced nutrition for Chinese women preparing for pregnancy to avoid adverse pregnancy outcomes.

## Figures and Tables

**Table 1:** Baseline characteristics of the study participants.

Characteristics	Total (*n* = 1546)	Prepregnancy BMI				*P*
		Underweight (*n* = 154)	Normal (*n* = 1049)	Overweight (*n* = 238)	Obese (*n* = 105)	
Age, years (mean ± SD)	30.77 ± 3.30	30.03 ± 2.90	30.55 ± 3.11	31.55 ± 3.69	31.08 ± 3.57	<0.001
History of pregnancy, *n* (%)						0.240
Nulliparous	125 (8.09)	17 (11.04)	83 (7.91)	14 (5.88)	11 (10.48)	
Yes	1421 (91.91)	137 (88.96)	966 (92.09)	224 (94.12)	94 (89.52)	
Conception method, *n* (%)						<0.001
ART	130 (8.46)	7 (4.61)	72 (6.90)	32 (13.50)	19 (18.27)	
Spontaneous pregnancy	1407 (91.54)	145 (95.39)	972 (93.10)	205 (86.50)	85 (81.73)	
Number of pregnancies, *n* (%)						0.291
Single	1499 (97.27)	152 (99.35)	1016 (97.13)	229 (96.22)	102 (98.08)	
Multiple	42 (2.73)	1 (0.65)	30 (2.87)	9 (3.78)	2 (1.92)	

Note: prepregnancy BMI groups were defined underweight < 18.5 kg/m2, normal 18.5-23.9 kg/m2, overweight 24-27.9 kg/m2, and obese ≥ 28 kg/m2. *P* values represent the significance in the difference of the variable distribution among underweight, normal, overweight, and obese groups, with *P* < 0.05 as statistically significant difference. Abbreviations: SD: standard deviation; ART: assisted reproductive technology.

**Table 2:** The association between prepregnancy BMI and pregnancy outcomes, unadjusted logistic regression model.

Pregnancy outcomes	Unadjusted logistic regression model			
	Underweight (*n* = 154)	Normal (*n* = 1049)	Overweight (*n* = 238)	Obese (*n* = 105)
Gestational diabetes	0.679 (0.432-1.067)	Ref	1.003 (0.715-1.407)	2.838 (1.881-4.282)^∗∗∗^
PPROM	1.757 (1.210-2.549)^∗∗^	Ref	0.807 (0.559-1.167)	0.857 (0.510-1.440)
Anemia	1.225 (0.800-1.875)	Ref	0.982 (0.674-1.430)	0.347 (0.159-0.760)^∗∗^
Postpartum hemorrhage	0.784 (0.384-1.597)	Ref	1.548 (0.969-2.474)	1.329 (0.665-2.654)
Preeclampsia	0.354 (0.047-2.666)	Ref	1.402 (0.554-3.549)	4.472 (1.908-10.483)^∗∗^

Note: prepregnancy BMI groups were defined underweight < 18.5 kg/m^2^, normal 18.5-23.9 kg/m^2^, overweight 24-27.9 kg/m^2^, and obese ≥ 28 kg/m^2^. ^∗^*P* < 0.05, ^∗∗^*P* < 0.01, and ^∗∗∗^*P* < 0.001. Abbreviations: BMI: body mass index; PPROM: preterm premature rupture of membranes.

**Table 3:** The association between prepregnancy BMI and pregnancy outcomes, adjusted logistic regression model.

Pregnancy outcomes	Adjusted logistic regression model			
	Underweight (*n* = 154)	Normal (*n* = 1049)	Overweight (*n* = 238)	Obese (*n* = 105)
Gestational diabetes	0.766 (0.485-1.212)	Ref	0.990 (0.698-1.406)	2.758 (1.782-4.269)^∗∗∗^
PPROM	1.944 (1.329-2.845)^∗∗^	Ref	0.841 (0.572-1.236)	0.963 (0.562-1.653)
Anemia	1.201 (0.778-1.855)	Ref	0.974 (0.664-1.428)	0.345 (0.156-0.760)^∗∗^
Postpartum hemorrhage	0.745 (0.350-1.586)	Ref	1.451 (0.879-2.398)	1.221 (0.584-2.555)
Preeclampsia	0.350 (0.046-2.641)	Ref	1.340 (0.523-3.436)	3.512 (1.408-8.762)^∗∗^

Note: prepregnancy BMI groups were defined underweight < 18.5 kg/m^2^, normal 18.5-23.9 kg/m^2^, overweight 24-27.9 kg/m^2^, and obese ≥ 28 kg/m^2^. ^∗^*P* < 0.05, ^∗∗^*P* < 0.01, and ^∗∗∗^*P* < 0.001. All analyses were adjusted for age and conception method. Abbreviations: BMI: body mass index; PPROM: preterm premature rupture of membranes.

**Table 4:** Effects of prepregnancy BMI on pregnancy outcomes.

Pregnancy outcomes	Underweight (*n* = 154)	Normal (*n* = 1049)	Overweight (*n* = 238)	Obese (*n* = 105)
Gestational diabetes	0.778 (0.491-1.231)	Ref	0.996 (0.702-1.414)	2.726 (1.757-4.231)^∗∗∗^
PPROM	1.902 (1.296-2.790)^∗∗^	Ref	0.845 (0.575-1.243)	0.976 (0.568-1.676)
Anemia	1.218 (0.788-1.882)	Ref	0.973 (0.664-1.428)	0.299 (0.128-0.698)^∗∗^
Postpartum hemorrhage	0.759 (0.356-1.616)	Ref	1.444 (0.874-2.387)	1.254 (0.599-2.628)
Preeclampsia	0.365 (0.048-2.757)	Ref	1.329 (0.517-3.416)	3.766 (1.504-9.427)^∗∗^

Note: prepregnancy BMI groups were defined underweight < 18.5 kg/m^2^, normal 18.5-23.9 kg/m^2^, overweight 24-27.9 kg/m^2^, and obese ≥ 28 kg/m^2^. ^∗^*P* < 0.05, ^∗∗^*P* < 0.01, and ^∗∗∗^*P* < 0.001. All analyses were adjusted for age, conception method, and the number of pregnancies. Abbreviations: BMI: body mass index; PPROM: preterm premature rupture of membranes.

**Table 5:** Odds ratio for pregnancy outcomes by prepregnancy BMI categories.

Pregnancy outcomes	Underweight (*n* = 154)	Normal (*n* = 1049)	Overweight (*n* = 238)	Obese (*n* = 105)
Gestational diabetes^a^	0.783 (0.495-1.240)	Ref	0.993 (0.699-1.410)	2.649 (1.701-4.126)^∗∗∗^
PPROM^b^	1.864 (1.269-2.737)^∗∗^	Ref	0.850 (0.577-1.250)	1.090 (0.630-1.889)
Anemia^c^	1.218 (0.788-1.884)	Ref	0.971 (0.662-1.424)	0.300 (0.128-0.704)^∗∗^
Postpartum hemorrhage^d^	0.728 (0.338-1.565)	Ref	1.460 (0.876-2.432)	1.468 (0.684-3.152)
Preeclampsia^e^	0.368 (0.049-2.781)	Ref	1.319 (0.512-3.394)	3.654 (1.420-9.404)^∗∗^

Note: ^a^model adjusted for age, conception method, the number of pregnancies, anemia, and preeclampsia. ^b^Model adjusted for age, conception method, the number of pregnancies, gestational diabetes, anemia, and preeclampsia. ^c^Model adjusted for age, conception method, the number of pregnancies, gestational diabetes, and preeclampsia. ^d^Model adjusted for age, conception method, the number of pregnancies, gestational diabetes, anemia, PPROM, and preeclampsia. ^e^Model adjusted for age, conception method, the number of pregnancies, gestational diabetes, and anemia. Abbreviations: BMI: body mass index; PPROM: preterm premature rupture of membranes.

## Data Availability

The data used to support the findings of this study are available from the corresponding author upon request.
